# Pre- and Postoperative Models for Prediction of Recurrence in Non-B, Non-C Hepatocellular Carcinoma

**DOI:** 10.3389/fonc.2021.612588

**Published:** 2021-02-18

**Authors:** Kongying Lin, Qizhen Huang, Lei Wang, Jianxing Zeng, Zongren Ding, Hongzhi Liu, Jun Fu, Pengfei Guo, Zhenwei Chen, Yongyi Zeng, Weiping Zhou, Jingfeng Liu

**Affiliations:** ^1^ Department of Hepatopancreatobiliary Surgery, Mengchao Hepatobiliary Hospital of Fujian Medical University, Fuzhou, China; ^2^ Department of Radiation Oncology, Mengchao Hepatobiliary Hospital of Fujian Medical University, Fuzhou, China; ^3^ Department of Radiation Oncology, Fujian Medical University Cancer Hospital, Fujian Cancer Hospital, Fuzhou, China; ^4^ The Big Data Institute of Southeast Hepatobiliary Health Information, Mengchao Hepatobiliary Hospital of Fujian Medical University, Fuzhou, China; ^5^ The Third Department of Hepatic Surgery, Eastern Hepatobiliary Surgery Hospital, Second Military Medical University, Shanghai, China

**Keywords:** non-B non-C hepatocellular carcinoma, resection, recurrence, inflammation, nomogram

## Abstract

**Background and Aims:**

The incidence of non-B, non-C hepatocellular carcinoma (NBNC-HCC) is increasing. Like in hepatitis B virus (HBC)/HCV-associated HCC, treatment of NBNC-HCC after resection is challenging due to its high recurrence rate. However, few studies on the recurrence of NBNC-HCC have been published in the past decades. Hence, we aimed to investigate the risk factors for recurrence of NBNC-HCC and construct pre- and postoperative prognostic models for predicting recurrence in these patients who underwent curative resection.

**Methods:**

We retrospectively analyzed 608 patients who underwent liver resection for NBNC-HCC. A multivariate Cox proportional hazard regression analysis was conducted to identify the independent risk factors of recurrence, based on which the prediction nomogram models were constructed and validated. The predictive performance of the models was assessed using the concordance index, time-dependent receiver operating characteristic curve, prediction error cure, and calibration curve. To facilitate clinical use, we stratified the patients into three distinct risk groups based on the score of the models. The cutoff scores of the models were determined by a survival tree analysis.

**Results:**

Multivariable analysis identified neutrophil-to-lymphocyte ratio, alpha fetoprotein, tumor number, and tumor diameter as independent preoperative risk factors for recurrence. In addition to these variables, microvascular invasion was an independent postoperative risk factor for recurrence. The pre- and postoperative nomograms were constructed based on these variables. The C-index of the pre- and postoperative nomograms was 0.689 and 0.702 in the training cohort, 0.682 and 0.688 in the validation cohort, respectively, which were both higher than those of the conventional Barcelona Clinic Liver Cancer (BCLC) and 8^th^ edition of the American Joint Committee on Cancer (AJCC^8th^) staging systems. In addition, the pre- and postoperative nomograms could also re-stratify patients with BCLC stage 0/A or AJCC^8th^ stage IA/IB/II into distinct risk groups.

**Conclusions:**

We constructed pre- and postoperative prognostic models for predicting recurrence in patients with NBNC-HCC who underwent curative resection. They can play a supplementary role to the traditional staging system.

## Introduction

Hepatocellular carcinoma (HCC) is the fifth most common cancer and third leading cause of cancer-related deaths worldwide (1). Chronic infections with hepatitis B (HBV) and C viruses (HCV) are the prominent etiological factors for HCC. However, with the adoption of HBV immunization programs and control of HCV transmission, the incidence of HBV\HCV-associated HCC has decreased in recent years (2, 3). The number of patients with HCC who are seronegative for both HBV and HCV, so called “NBNC-HCC,” is gradually increasing (4, 5).

Due to the shortage of donor organs, surgical resection remains the main treatment strategy for patients with HCC who have a good liver function and resectable tumors (6). However, long-term survival after surgery remains unsatisfactory due to the high incidence of tumor recurrence (7). Owing to the heterogeneity of HCC, some highly selected patients may benefit from a prognostic prediction model, well-selected therapeutic assignment, and strict postoperative monitoring (8, 9). There are several studies on constructed prognostic models for HCC; however, very few studies have specifically focused on the recurrence of patients with NBNC-HCC. Considering the different clinical manifestations and prognostic outcomes of NBNC-HCC and viral-associated HCC, the study of its risk factors and establishment of prognostic models may provide important insight into novel strategies for the treatment and postoperative monitoring of NBNC-HCC.

Host inflammatory response to cancer and tumor-mediated systemic inflammation promote migration, invasion, and metastasis of malignant cells (10, 11); they are prognostic factors for HCC (12). Recent studies have demonstrated that preoperative inflammatory indices, such as the neutrophil-to-lymphocyte ratio (NLR) (13), neutrophil times γ-glutamyl transpeptidase-to-lymphocyte ratio (NγLR) (14), platelet-to-lymphocyte ratio (PLR) (15), prognostic nutritional index (PNI) (16), aspartate aminotransferase-to-neutrophil ratio (ANRI) (17), γ-glutamyl transpeptidase-to-platelet ratio (GPR) (18), aspartate aminotransferase-to-lymphocyte ratio index (ALRI) (19) and platelet times neutrophil-to-lymphocyte ratio (PNLR) (20) are independent prognostic factors for patients with HCC who have undergone liver resection and have been used in the construction of several prognosis prediction models; however, the relationship between these inflammatory indexes and prognosis of NBNC-HCC, and the inflammatory index with the highest prognostic significance remain unclear.

In our study, we aimed to: (1) investigate the relationship between inflammatory indexes and recurrence of NBNC-HCC, (2) investigate the pre- and postoperative risk factors for recurrence of NBNC-HCC, and (3) construct a pre- and postoperative nomogram model for the prediction of recurrence of NBNC-HCC.

## Materials and Methods

### Patients

Data from patients diagnosed with NBNC-HCC who underwent hepatectomy as a primary anti-cancer therapy between April 28, 2008 and December 30, 2015 were extracted from the primary liver cancer big data (PLCBD) (21, 22). In this study, patients with HCC who were seronegative for hepatitis B surface antigen, HBV- deoxyribonucleic acid (DNA), hepatitis C antibody, and HCV-RNA test were considered patients with NBNC-HCC (23). All data in this study were verified by three independent researchers, and the study was approved by the institutional ethics committee of Mengchao Hepatobiliary Hospital of Fujian Medial University.

Patients with Child Pugh A or B7 liver function, no extrahepatic metastasis, no macroscopic vascular invasion, and who underwent R0 resection (complete removal of all detectable tumor nodes with tumor-free margins confirmed by histological examination) were included. Patients who underwent palliative tumor resection or any preoperative anti-HCC therapy, with a history of other cancers, or incomplete clinical data were excluded.

### Preoperative Assessment, Hepatectomy, and Follow-Up

All patients underwent routine preoperative examinations, which included hepatitis B and C immunology, HBV-DNA load, alpha-fetoprotein (AFP) levels, liver and kidney function examinations, chest radiography, abdominal ultrasonography, contrast-enhanced computed tomography, and magnetic resonance imaging. HCC was diagnosed according to the practice guidelines recommended by the American Association for the Study of Liver Diseases (24). The choice of performing anatomical or partial hepatectomy is commonly based on the patient’s liver function status, tumor number, and location. In simple terms, anatomical hepatectomy was preferentially performed for patients with a good liver function and tumors located within a segment, sector, and hemiliver. Partial hepatectomy was performed for tumors that were peripherally located or patients with unsatisfactory liver function. Intraoperative liver ultrasonography was routinely used to ensure that all detectable tumors were completely removed. The follow-up of patients after discharge was performed in the outpatient clinic. The follow-up program and diagnostic criteria for tumor recurrence are reported in a previous study (9).

### Clinicopathologic Variables

For hematological investigations, we used the results of the most recent test that was performed within 15 days prior to surgery. The formulae of the indices (NLR, PLR, GPR, PNLR, PNI, ANRI, ALRI, DNLR, and NγLR) are reported in a previous study (14, 18, 25–27). The preoperative imaging data, including tumor number and diameter was obtained from contrast-enhanced Computed Tomography or contrast-enhanced magnetic resonance imaging. Tumor diameter was the diameter of the largest tumor. Histologic grading of the tumor was done according to the Edmondson-Steiner classification. The definition of microvascular invasion (MVI) from a previous study was used (28). The pathological review of all resected specimens was carried out independently by two pathologists.

### Statistical Analysis

Categorical variables were expressed as number (percentage) and compared using the χ2 test or Fisher’s exact test. Continuous variables were expressed as mean (standard deviation SD) and compared using the Student t test or the Mann–Whitney U test. Univariate and multivariate Cox proportional hazard regression analyses were performed to determine the independent prognostic factors for recurrence. Clinical variables considered to be potentially relevant (p<0.05 in univariate analyses) were entered in the multivariate Cox proportional hazard regression analyses, and the final independent risk factors for recurrence were identified by the multivariable analyses with stepwise backward selection method.

The nomograms were constructed based on the results of multivariable analyses of recurrence in the training cohort. The R code used for construction of nomogram was shown in the supporting information. The preoperative nomogram was developed on the available preoperative clinicopathologic data. The postoperative nomogram was based on all available clinicopathologic variables. The predictive performance of the nomograms was measured using Harrell’s concordance index (C-index), time-dependent areas under the receiver operating characteristic curve (tdAUC), prediction error curve, and calibration plot (29, 30). The cutoff of models was determined by a survival tree analysis (29). The cumulative recurrence between each risk group was assessed and tested using Kaplan-Meier curves and the log-rank test, respectively. All statistical tests were two-tailed, and P < 0.05 was considered statistically significant. Statistical analyses were performed using SPSS version 20.0 and R version 3.0 (http://www.r-project.org/); the R packages of “table1,” “rms,” “CsChange,” “survminer,” “survival,” “pec,” “riskRegression,” “timeROC,” and “party” were used.

## Results

### Patients’ Characteristics

A total of 608 eligible patients were included in the study. They were randomly divided into a training cohort (n=456) and validation cohort (n=152) in a 3:1 ratio. A comparison of the clinicopathologic characteristics between the two cohorts is shown in **Table 1**. There were no significant differences in the clinicopathologic features between the two cohorts. The mean age was 59.2 ± 11.2 years and 58.3 ± 11.7 years in the training and validation cohorts, respectively. Most of the patients were male (85.5–87.5%), and few patients had cirrhotic livers (32.9–35.5%). The mean tumor diameter was 7.06 ± 3.99 cm and 6.80 ± 4.12 cm in the training and validation cohorts, respectively. Most of the patients harbored solitary tumors (86.2–83.6%).

**Table 1:** Baseline clinical characteristics of patients.

	Training cohort	Validation cohort	P-value
	(n=456)	(n=152)	
**Age,** Mean (SD), years	59.2 (11.2)	58.3 (11.7)	0.402
**Gender**			
Female	66 (14.5%)	19 (12.5%)	0.636
Male	390 (85.5%)	133 (87.5%)	
**Alcohol consumption**			0.407
No	321 (70.4%)	113 (74.3%)	
Yes	135 (29.6%)	39 (25.7%)	
**Cigarette smoking**			0.065
No	278 (61.0%)	106 (69.7%)	
Yes	178 (39.0%)	46 (30.3%)	
**Hypertension**			0.959
Absent	321 (70.4%)	108 (71.1%)	
Present	135 (29.6%)	44 (28.9%)	
**Diabetes**			0.126
Absent	389 (85.3%)	121 (79.6%)	
Present	67 (14.7%)	31 (20.4%)	
**Fatty liver**			0.501
Absent	403 (88.4%)	138 (90.8%)	
Present	53 (11.6%)	14 (9.2%)	
**Cirrhosis**			0.620
Absent	306 (67.1%)	98 (64.5%)	
Present	150 (32.9%)	54 (35.5%)	
**BCLC staging system**			0.684
0	9 (2.0%)	3 (2.0%)	
A	396 (86.8%)	128 (84.2%)	
B	51 (11.2%)	21 (13.8%)	
**AJCC staging system^8th^**			0.961
IA	9 (2.0%)	3 (2.0%)	
IB	295 (64.7%)	96 (63.2%)	
II	105 (23.0%)	35 (23.0%)	
IIIA	47 (10.3%)	18 (11.8%)	
**HbcAb**			0.370
Negative	57 (12.5%)	24 (15.8%)	
Positive	399 (87.5%)	128 (84.2%)	
**WBCs**, Mean (SD), 10^9^/L	5.85 (1.92)	5.67 (1.85)	0.320
**RBCs**, Mean (SD), 10^9^/L	4.55 (0.57)	4.52 (0.53)	0.559
**Hb**, Mean (SD), g/L	138.27 (17.47)	136.80 (16.15)	0.343
**Neutrophil Count**, Mean (SD), 10^9^/L	3.68 (1.62)	3.53 (1.48)	0.305
**Lymphocyte Count**, Mean (SD), 10^9^/L	1.66 (0.61)	1.64 (0.61)	0.640
**Platelets**, Mean (SD), 10^9^/L	194.37 (75.32)	194.85 (85.53)	0.951
**Total bilirubin**, Mean (SD), umol/L	13.4 (5.29)	13.2 (5.10)	0.604
**Albumin**, Mean (SD), g/L	42.7 (3.71)	42.1 (3.60)	0.069
**ALT**, Mean (SD), U/L	31.1 (24.9)	29.7 (24.6)	0.538
**AST**, Mean (SD), U/L	34.0 (27.0)	30.7 (26.1)	0.186
**GGT**, Mean (SD), U/L	76.3 (111)	89.7 (169)	0.360
**NLR**	2.47 (1.43)	2.36 (1.18)	0.371
**PLR**	127.27 (59.35)	127.87 (59.36)	0.914
**PNI**	51.00 (5.31)	50.24 (5.24)	0.127
**ANRI**	10.63 (9.47)	9.99 (8.50)	0.436
**ALRI**	23.89 (22.33)	21.53 (17.55)	0.182
**PNLR**	491.55 (419.79)	471.33 (360.71)	0.567
**GPR**	0.887 (1.40)	1.170 (2.79)	0.232
**NγLR**	195.32 (341.11)	185.94 (261.38)	0.724
**DNLR**	1.76 (1.44)	1.75 (0.75)	
**AFP**, ng/ml			0.482
≤20	236 (51.8%)	73 (48.0%)	
>20	220 (48.2%)	79 (52.0%)	
**Intraoperative blood transfusion**			0.882
No	406 (89.0%)	134 (88.2%)	
Yes	50 (11.0%)	18 (11.8%)	
Tumor size^*^, Mean (SD), cm	6.92 (3.91)	6.68 (4.07)	0.522
Tumor number^*^			0.380
Solitary	396 (86.8%)	127 (83.6%)	
Multiple	60 (13.2%)	25 (16.4%)	
**Tumor size**, Mean (SD), cm	7.06 (3.99)	6.80 (4.12)	0.501
**Tumor number**			0.506
Solitary	393 (86.2%)	127 (83.6%)	
Multiple	63 (13.8%)	25 (16.4%)	
**MVI**			0.709
Absent	339 (74.3%)	110 (72.4%)	
Present	117 (25.7%)	42 (27.6%)	
**Satellite nodules**			0.587
Absent	304 (66.7%)	97 (63.8%)	
Present	152 (33.3%)	55 (36.2%)	
**Tumor capsule**			0.272
Complete	92 (20.2%)	39 (25.7%)	
Incomplete	268 (58.8%)	79 (52.0%)	
None	96 (21.1%)	34 (22.4%)	
**Edmondson-Steiner classification**			0.727
I/II	91 (20.0%)	33 (21.7%)	
III/IV	365 (80.0%)	119 (78.3%)	

BCLC, Barcelona Clinic Liver Cancer staging system; AJCC, American Joint Committee on Cancer; HBcAb, hepatitis B virus core antibody; RBCs, red blood cells; WBCs, white blood cells; Hb, hemoglobin; ALT, alanine aminotransferase; AST, aspartate aminotransferase; GGT, gamma-glutamyl transpeptidase; NLR, neutrophil to lymphocyte ratio; PLR, platelet-to-lymphocyte ratio; PNI, prognostic nutritional index; ANRI, AST to neutrophil ratio index; ALRI, AST to Lymphocyte ratio index; PNLR, platelet–neutrophil–lymphocyte ratio; GPR, gamma-glutamyl transpeptidase (GGT) to platelet ratio; NγLR, neutrophil times γ-glutamyl transpeptidase to lymphocyte ratio; DNLR, derived neutrophil to lymphocyte ratio; AFP, alpha fetoprotein; MVI, microvascular invasion.

*Preoperative images were obtained from contrast-enhanced Computed Tomography or contrast-enhanced magnetic resonance imaging.

## Postoperative Prognosis

The median follow-up period was 44.3 (range, 2.1–114.7) months and 45.3 months (range, 1.9–114.7) months in the training cohort and validation cohort, respectively. In the training cohort, the postoperative 1-, 3-, and 5-year overall survival (OS) rates were 91.7, 72.8, and 57.4%, respectively, and the corresponding cumulative recurrence rates were 24.2, 37.5, and 48.6%, respectively. In the validation cohort, the postoperative 1-, 3-, and 5-year OS rates were 87.2, 67.7, and 54.5%, respectively, and the corresponding cumulative recurrence rates were 28.8, 43.3, and 48.0%, respectively.

### Construction of Pre- and Postoperative Nomograms for Prediction of Recurrence

All the variables shown in **Table 1** were included in the univariate and multivariate Cox proportional hazard regression analyses to identify independent risk factors for recurrence. Results of the univariate analysis are shown in **Table 2**. On univariate analysis, there was a significant relationship between six inflammatory indexes (NLR, PLR, ALRI, PNLR, NγLR, and DNLR) and recurrence. Other significant variables were neutrophil count, lymphocyte count, aspartate aminotransferase, AFP, tumor diameter, tumor number, MVI, and satellite nodules.

**Table 2:** Univariate Cox regression analysis of risk factors of Recurrence in the training cohort.

Variable	B	SE	HR(95%CI)	P-value
**Age**, years	−0.003	0.006	0.997(0.985–1.009)	0.609
**Gender**, Male versus Female	−0.040	0.205	0.961(0.643–1.436)	0.846
**Alcohol consumption**, Yes versus No	0.084	0.159	1.088(0.797–1.485)	0.596
**Cigarette smoking**, Yes versus No	0.054	0.148	1.055(0.789–1.412)	0.716
**Hypertension**, present versus absent	0.070	0.157	1.072(0.789–1.458)	0.657
**Diabetes**, present versus absent	0.004	0.205	1.004(0.672–1.500)	0.984
**Fatty liver**, present versus absent	−0.234	0.242	0.791(0.492–1.272)	0.333
**Cirrhosis**, present versus absent	0.172	0.151	1.188(0.883–1.598)	0.255
**HbcAb**, Positive versus Negative	0.127	0.227	1.136(0.727–1.774)	0.576
**WBCs**, 10^9^/L	0.059	0.033	1.060(0.994–1.131)	0.074
**RBCs**, 10^9^/L	0.074	0.128	1.076(0.837–1.384)	0.566
**Hb,** g/L	−0.003	0.004	0.997(0.989–1.005)	0.450
**Neutrophil count**, 10^9^/L	0.104	0.031	1.110(1.044–1.180)	0.001
**Lymphocyte count**, 10^9^/L	−0.322	0.127	0.725(0.565–0.930)	0.011
**Platelets**, 10^9^/L	0.001	0.001	1.001(0.999–1.003)	0.387
**Total bilirubin**, umol/L	0.003	0.014	1.003(0.976–1.031)	0.835
**Albumin**, g/L	0.004	0.020	1.004(0.966–1.043)	0.850
**ALT**, U/L	0.002	0.003	1.002(0.997–1.007)	0.426
**AST**, U/L	0.004	0.002	1.004(1.001–1.008)	0.025
**GGT**, U/L	0.001	0.001	1.001(0.999–1.002)	0.329
**NLR**	0.187	0.038	1.206(1.120–1.299)	<0.001
**PLR**	0.003	0.001	1.003(1.001–1.005)	0.002
**PNI**	−0.018	0.014	0.982(0.956–1.009)	0.187
**ANRI**	0.007	0.006	1.007(0.994–1.019)	0.288
**ALRI**	0.007	0.002	1.007(1.002–1.012)	0.006
**PNLR**	0.0004	0.0001	1.0004(1.000–1.001)	<0.001
**GPR**	0.043	0.048	1.044(0.950–1.147)	0.372
**NγLR**	0.001	0.0001	1.001(1.000–1.001)	<0.001
**DNLR**	0.176	0.071	1.193(1.037–1.372)	0.014
**AFP**, ng/ml, >20 versus ≤20	0.444	0.146	1.558(1.171–2.074)	0.002
**Intraoperative blood transfusion**, Yes versus No	0.374	0.219	1.454(0.947–2.234)	0.087
Tumor size^*^, cm	0.089	0.017	1.093(1.057–1.130)	<0.001
Tumor number^*^, Multiple versus Solitary	1.301	0.172	3.673(2.623–5.145)	<0.001
**Tumor size**, cm	0.088	0.016	1.091(1.057–1.127)	<0.001
**Tumor number**, Multiple versus Solitary	1.274	0.169	3.576(2.566–4.983)	<0.001
**MVI**, Present versus Absent	0.726	0.153	2.067(1.531–2.792)	<0.001
**Satellite nodules**, Present versus Absent	0.746	0.147	2.109(1.582–2.811)	<0.001
**Tumor capsule**				0.345
Complete	Ref	Ref	Ref	Ref
Incomplete	0.200	0.194	1.221(0.835–1.785)	0.303
None	0.332	0.228	1.394(0.891–2.179)	0.146
**Edmondson-Steiner grading**, I/II versus III/IV	0.310	0.190	1.363 (0.940–1.977)	0.102

HBcAb, hepatitis B virus core antibody; RBCs, red blood cells; WBCs, white blood cells; Hb, hemoglobin; ALT, alanine aminotransferase; AST, aspartate aminotransferase; GGT, gamma-glutamyl transpeptidase; NLR, neutrophil to lymphocyte ratio; PLR, platelet-to-lymphocyte ratio; PNI, prognostic nutritional index; ANRI, AST to neutrophil ratio index; ALRI, AST to Lymphocyte ratio index; PNLR, platelet–neutrophil–lymphocyte ratio; GPR, gamma-glutamyl transpeptidase (GGT) to platelet ratio; NγLR, neutrophil times γ-glutamyl transpeptidase to lymphocyte ratio; DNLR, derived neutrophil to lymphocyte ratio; AFP, alpha fetoprotein; MVI, microvascular invasion; B, coefficient; SE, stand error; CI, confidence interval; HR, hazard ratio. *Preoperative images were obtained from contrast-enhanced Computed Tomography or contrast-enhanced magnetic resonance imaging.

To construct the preoperative model, presence of MVI and satellite nodules, which were recorded only postoperatively, were excluded, and the remaining 12 factors were entered into the multivariate Cox regression analysis using a stepwise method. All 14 risk factors that could be acquired preoperatively were entered in the multivariate analysis.

**Figure 1:**
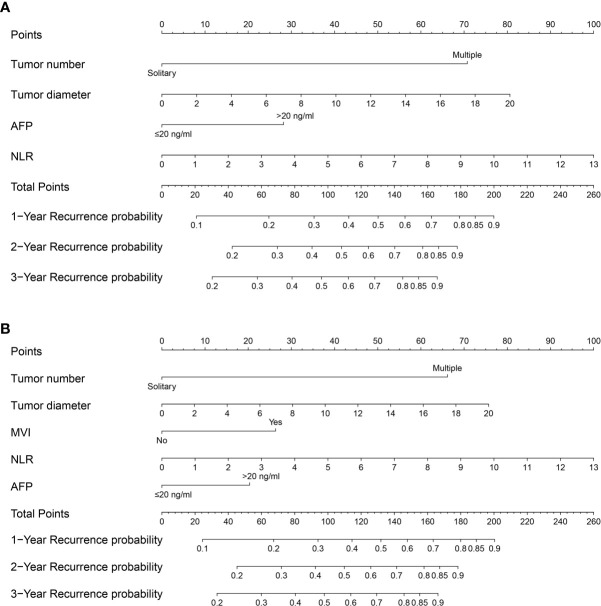
Nomogram for preoperative prediction **(A)** and postoperative prediction **(B)** of Recurrence for NBNCHCC patients who underwent hepatectomy.

Multivariate analyses revealed that multiple tumor number [hazard ratio with 95% confidence interval (CI)=3.381 (2.401–4.761), P<0.001], large tumor diameter [1.072 (1.035–1.111), P<0.001], elevated AFP levels [1.624 (1.218–2.166), P=0.001], and elevated NLR [1.142 (1.046–1.246), P=0.003] were independent preoperative risk factors for recurrence of NBNC-HCC (**Table 3**); Multivariate analysis of the postoperative variables revealed that multiple tumor number [3.187 (2.273–4.470), P<0.001], large tumor diameter [1.069 (1.033–1.106), P<0.001], presence of MVI [1.587 (1.153–2.186), P=0.005], elevated AFP levels [1.428 (1.057–1.930), P=0.02], and elevated NLR [1.144 (1.048–1.250), P=0.003] were independent postoperative risk factors for recurrence of NBNC-HCC (**Table 4**). These independent risk factors were then used to build the pre- and postoperative nomogram models (**Figures 1A, B**).

**Table 3:** Multivariate Cox regression analysis of risk factors of Recurrence based on Preoperative data of the training cohort.

Variable	B	SE	HR(95%CI)	P-value
Tumor number^*^, Multiple versus Solitary	1.218	0.175	3.381(2.401–4.761)	<0.001
Tumor size^*^, cm	0.069	0.018	1.072(1.035–1.111)	<0.001
**AFP**, ng/ml, >20 versus ≤20	0.485	0.147	1.624(1.218–2.166)	0.001
**NLR**	0.132	0.045	1.142(1.046–1.246)	0.003

NLR, neutrophil to lymphocyte ratio; AFP, alpha fetoprotein; B, coefficient; SE, stand error; CI, confidence interval; HR, hazard ratio.

*Preoperative images were obtained from contrast-enhanced Computed Tomography or contrast-enhanced magnetic resonance imaging.

**Table 4:** Multivariate Cox regression analysis of risk factors of Recurrence based on Postoperative data of the training cohort.

Variable	B	SE	HR(95%CI)	P-value
**Tumor number**, Multiple versus Solitary	1.159	0.173	3.187(2.273–4.470)	<0.001
**Tumor size**, cm	0.066	0.017	1.069(1.033–1.106)	<0.001
**MVI**, Present versus Absent	0.462	0.163	1.587(1.153–2.186)	0.005
**AFP**, ng/ml, >20 versus ≤20	0.357	0.154	1.428(1.057–1.930)	0.02
**NLR**	0.135	0.045	1.144(1.048–1.250)	0.003

NLR, neutrophil to lymphocyte ratio; AFP, alpha fetoprotein; MVI, microvascular invasion;

B, coefficient; SE, stand error; CI, confidence interval; HR, hazard ratio.

### Performance of the Pre- and Postoperative Nomograms in Recurrence Prediction

In the training and validation cohorts, both nomograms had a satisfactory performance in recurrence prediction. The C-index of the preoperative nomogram in the training and validation cohorts was 0.689 (95% CI, 0.651–0.728) and 0.682 (0.618–0.746), respectively, which were significantly higher than the currently used Barcelona Clinic Liver Cancer (BCLC) staging system (31) [0.593 (0.564–0.622), p<0.001; 0.593 (0.544–0.641), p=0.001] and 8^th^ edition of the American Joint Committee on Cancer (AJCC) staging system [0.645 (0.608–0.682), p=0.018; 0.624 (0.562–0.687), p=0.024]. For the postoperative nomogram model, the C-index values were 0.702 (0.664–0.739) in the training cohort and 0.688 (0.622–0.753) in the validation cohort, which were greater than those of the BCLC (p<0.001, p=0.001, respectively) and 8^th^ edition of the AJCC (p<0.001, p=0.002, respectively) staging systems.

**Figure 2:**
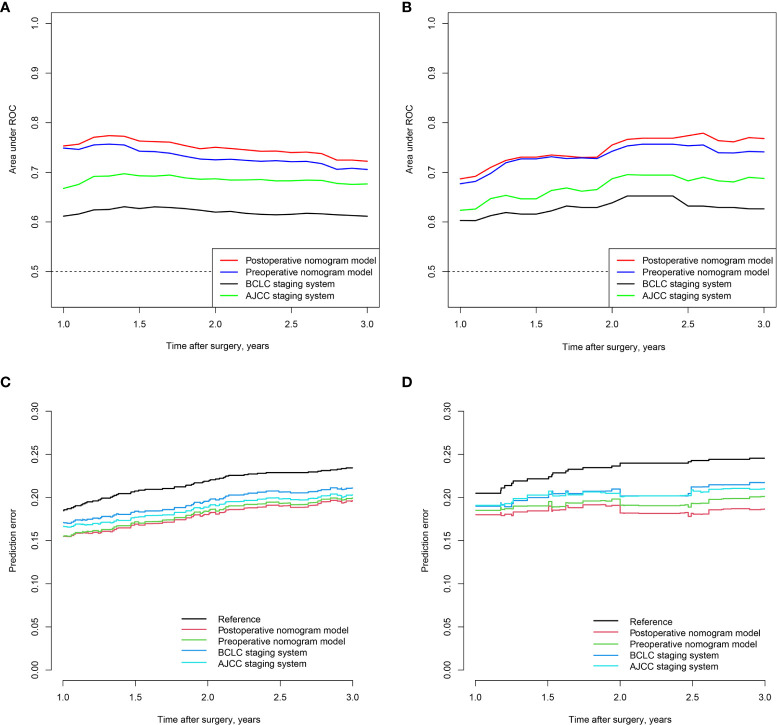
Time-dependent AUC of pre- and postoperative of nomograms in the training **(A)** and validation **(B)** cohorts; prediction error curve of pre- and postoperative nomogram models in the training **(C)** and validation cohorts **(D)**.

Time-dependent receiver operating characteristic curve analysis was also performed to assess the discriminative performance of the nomograms. For the preoperative nomogram model, the median tdAUCs for prediction of 1-, 2-, and 3 year recurrences were 0.725 (range, 0.706–0.749) in the training cohort and 0.741 (0.677–0.743) in the validation cohort. For the postoperative nomogram model, the corresponding tdAUC was 0.751 (range, 0.722–0.753) in the training cohort and 0.755 (range, 0.687–0.768) in the validation cohort. Both models had higher tdAUCs than those of the BCLC and AJCC staging systems (**Figures 2A, B**). In addition, the prediction error curve analysis was used to assess the overall performance of the models. The nomogram models had a lower prediction error rate than the conventional staging systems (**Figures 2C, D**).

**Figure 3:**
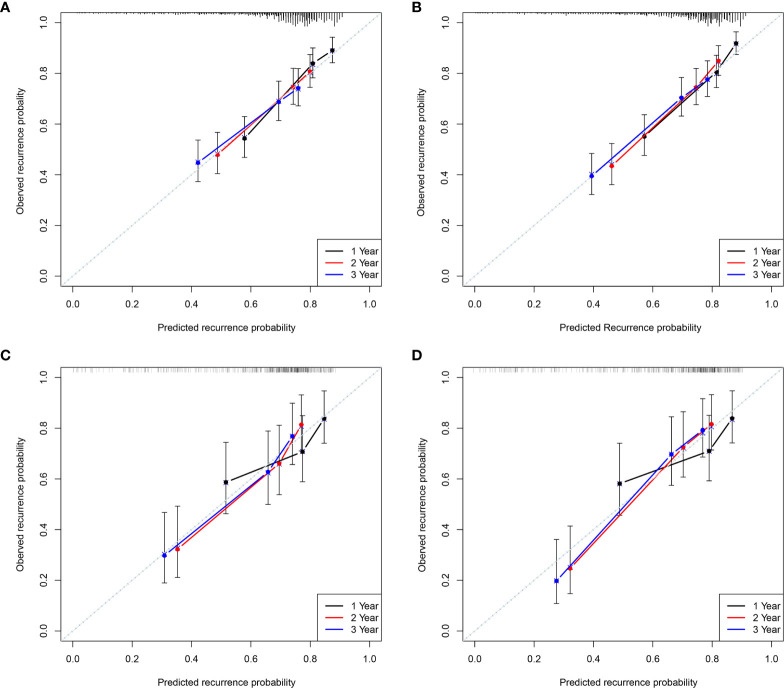
The calibration curves for predicting the 1, 2, and 3-year Recurrence by the Pre- and postoperative nomogram in the training **(A, B)** and validation cohorts **(C, D)**.

The calibration plots also displayed a good agreement between predictions of the pre- and postoperative nomogram models and the probability of 1-, 2-, and 3-year recurrence in the training and validation cohorts (**Figure 3**).

### Risk Stratification Based on the Nomograms’ Score

**Figure 4:**
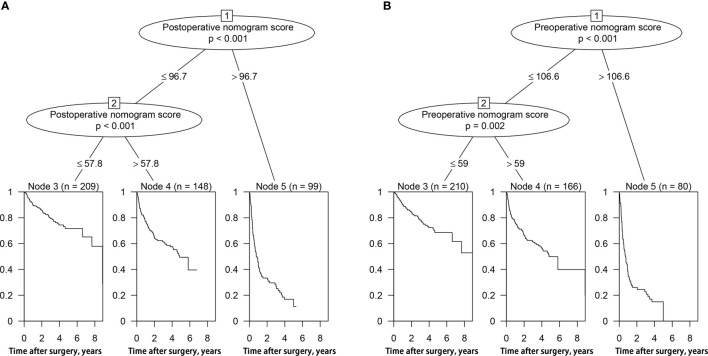
Survival tree analysis of best cut-off scores in the training cohort. **(A)** postoperative nomogram model; **(B)** preoperative nomogram model.

**Figure 5:**
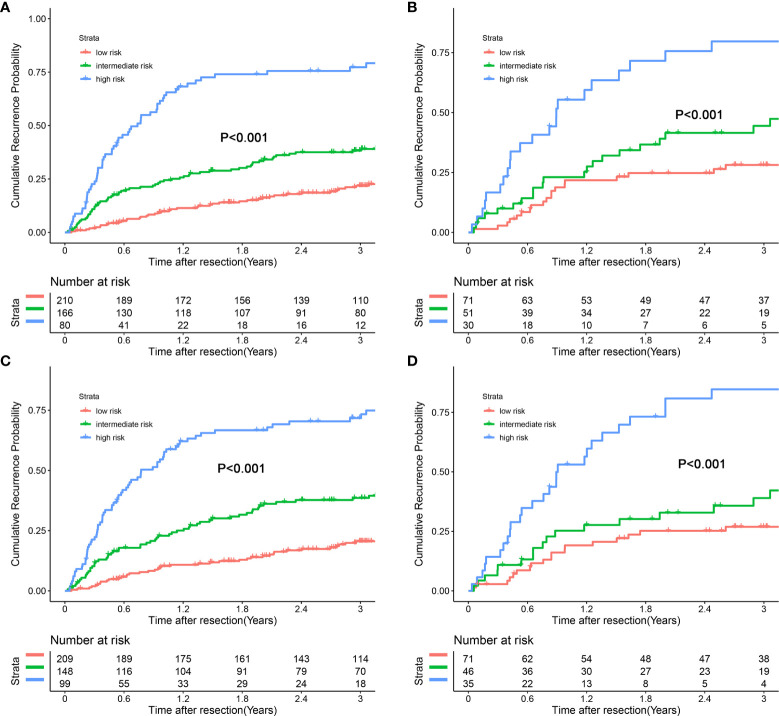
Kaplan-Meier plots for cumulative recurrence rate of risk subgroups defined by the nomograms model scores. **(A)** preoperative nomogram model, training cohort; **(B)** preoperative nomogram model, validation cohort; **(C)** postoperative nomogram model, training Cohort; **(D)** postoperative nomogram model, validation Cohort.

Each patient received an individualized risk score according to the individual scores calculated with the nomograms. We performed a survival tree analysis to determine the cutoff points of the risk score (**Figure 4**). Based on the cutoff points, the patients were stratified into three different risk subgroups (low-risk, intermediate-risk, and high-risk). The Kaplan-Meier analysis showed that the recurrence curves were widely separated among the three different risk groups in the training and validation cohorts, which further indicated that the nomograms had good discrimination ability for recurrence (**Figure 5**).

**Figure 6:**
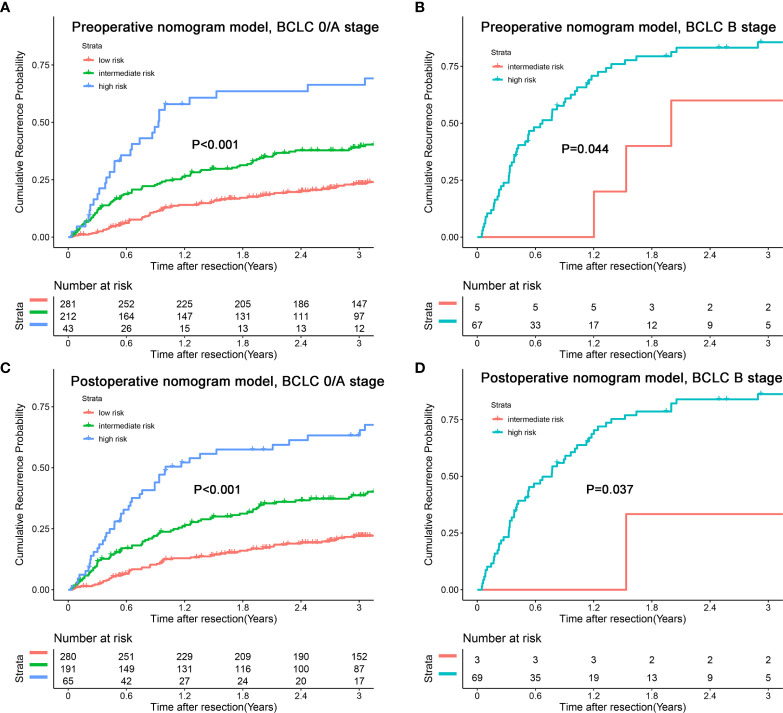
Kaplan-Meier plots for cumulative recurrence rate of risk subgroups defined by the nomograms model scores in different BCLC stage. **(A)** preoperative nomogram model, BCLC 0/A stage; **(B)** preoperative nomogram model, BCLC B stage; **(C)** postoperative nomogram model, BCLC 0/A stage; **(D)** postoperative nomogram model, BCLC B stage.

**Figure 7:**
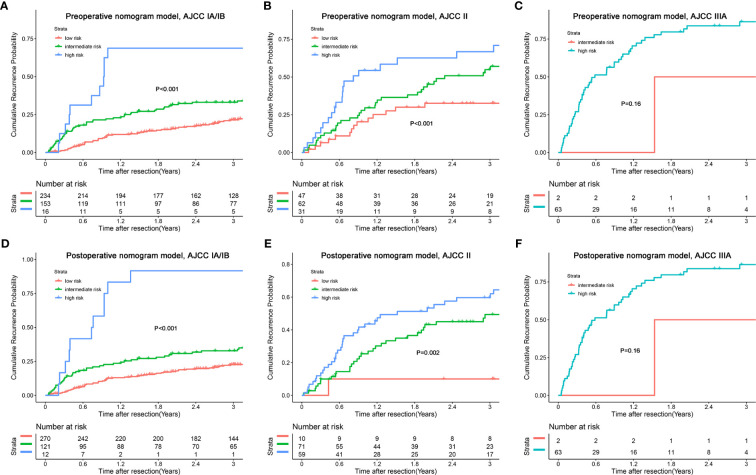
Kaplan-Meier plots for cumulative recurrence rate of risk subgroups defined by the nomograms model scores in different AJCC^8th^ stage. **(A)** preoperative nomogram model, AJCC IA/IB stage; **(B)** preoperative nomogram model, AJCC II stage; **(C)** preoperative nomogram model, AJCC IIIA stage; **(D)** postoperative nomogram model, AJCC IA/IB stage; **(E)** postoperative nomogram model, AJCC II stage; **(F)** postoperative nomogram model, AJCC IIIA stage.

In addition, we combined the models with the BCLC/AJCC^8th^ staging system and found that the models had a good re-stratification effect on the traditional staging systems. Because there were only 12 patients with BCLC stage 0 and AJCC stage IA tumors, these patients were incorporated into the group of patients with BCLC stage A or AJCC 8^th^ stage IB. As shown in **Figure 6**, both models could re-stratify the patients with different recurrence risks in BCLC stages 0/A (p<0.001, preoperative nomogram model; p<0.001, postoperative nomogram model) well. The re-stratification ability of the models persisted in patients with BCLC B stage (p=0.044, preoperative nomogram model; p=0.037, postoperative nomogram model), but given that only a small number of patients were in the high-risk group, the re-stratification ability of models in patients with BCLC B stage needed be validated in a further large sample cohort. For the AJCC^8th^ staging system, we found that the models distinguished patients with stages IA/IB (p<0.001, preoperative nomogram model; p<0.001, postoperative nomogram model), and II (p<0.001, preoperative nomogram model; p=0.002, postoperative nomogram model) well (**Figure 7**).

## Discussion

In this study, we reported significant independent risk factors for recurrence of NBNC-HCC, which included large tumor diameter, multiple tumor number, elevated AFP levels, elevated NLR, and presence of MVI. We also constructed pre- and postoperative nomograms for individualized prediction of recurrence in patients with NBNC-HCC who underwent curative resection. Both nomograms had a better predictive performance than the currently used BCLC and AJCC^8th^ staging systems. Additionally, according to the individualized scores assessed using the nomograms, patients in cohorts can be stratified into three risk groups. These two easy-to-apply graphical models will be valuable in preoperative treatment planning, adjuvant therapy implementation, postoperative monitoring, and designing of clinical trials based on prognostic stratification.

Tumor diameter and number are important prognostic factors in the BCLC staging system; however, these tumor factors are insufficient to reflect the malignant characteristics of HCC (32). Compared to the BCLC staging system, the AJCC^8th^ staging system included the presence of MVI as a stratification criterion; however, given that MVI is a pathological feature that can only be diagnosed after surgery, it limits the applicability of this system in preoperative clinical decision making. Moreover, both the BCLC and AJCC^8th^ staging systems were not developed specifically for the prediction of HCC recurrence. The nomograms in this study integrated the independent risk factors for recurrence of NBNC-HCC, including the above three tumor-associated factors; serum tumor biomarker, AFP; and inflammatory index, NLR; thus, making them more accurate recurrence predictors.

Our recurrence nomograms were able to re-stratify patients in the same traditional staging system stages, and thus can play a supplementary role to the traditional staging system. The preoperative nomogram in this study, which had a significantly better predictive performance than the BCLC staging system, may be an additional tool for surgeons to identify high-risk patients before operation; thus, it may be valuable in preoperative treatment planning [e.g., preoperative transarterial chemoembolization (TACE), widened surgical margin]. Owing to the lack of consensus on follow-up procedures for the postoperative diagnosis of recurrence of HCC (33), using the postoperative nomogram can help surgeons to design stricter follow-up procedures (e.g., reduced interval of follow-up and more high-end imaging tests) and postoperative adjuvant therapy for the high-recurrence risk patients. For the current results, it is difficult to specify which model is better for the recurrence prediction. In terms of the predictive performance of the models, the C-index and tdAUC of the postoperative model were higher than those of the preoperative model, and the prediction error curve also shows that the postoperative model is better than the preoperative model, although the difference was not obvious, the postoperative model may be superior to the preoperative model considering that the postoperative model includes the indicator of MVI, MVI is currently widely recognized as an independent risk factor for recurrence in HCC. However, when the preoperative model shows poor prognosis of patients and the postoperative model shows good prognosis, it may more proper to choose the preoperative model. After all, the cost of shortening the follow-up interval is much less than the cost of finding recurrence of HCC too late. Further prospective studies may be needed to distinguish which model has better predictive power.

In addition, we included an inflammatory index in this study. To the best of our knowledge, this is the first study to investigate the association between a system inflammatory index and recurrence in NBNC-HCC. In this study, we analyzed nine inflammatory indices and found that the NLR was an independent risk factor for recurrence in NBNC-HCC. High NLR has been reported to be a poor prognostic factor for recurrence and OS in several malignancies, including HCC (13, 34). Given that NLR consists of serum neutrophil count and lymphocyte count, elevation of NLR can be reviewed as elevated neutrophil and lymphocyte counts. Neutrophilia can promote cancer cell growth and progression by releasing angiogenic factors and inflammatory mediators (28, 35, 36). On the other hand, lymphocytes play an anti-cancer role in host immunity by inducing cytotoxicity and inhibiting proliferation, invasion, and migration of cancer cells; lymphopenia may weaken this anti-cancer effect (37–39). All these factors included in the NLR were adverse factors for HCC, which could possibly explain why NLR could be used to evaluate the recurrence probability of patients with HCC. However, the mechanism underlying the association between NLR and recurrence of NBNC-HCC remains unclear and needs further elucidation.

This study had some limitations. First, it was limited by its retrospective nature and limited sample size; thus, selection bias was unavoidable. A large sample, outer validation cohort, and prospective study are needed to validate the nomograms in future. Second, postoperative adjuvant therapy information, such as postoperative adjuvant TACE (PA-TACE), has not been included in our study. Given that PA-TACE may improve the prognostic outcome of high-risk patients (40, 41), whether high-risk patients assessed by these nomograms can benefit from PA-TACE is to be determined. Third, the prognostic value of postoperative inflammatory indexes or the changed indexes after post-operation compared to pre-operation in HCC have been recognized in some studies, but this study did not include because the data of some postoperative inflammatory indexes were missing. Last, the nomograms were generated using data of patients who underwent radical resection, which may not be applicable for patients receiving other therapies.

## Conclusion

In summary, we developed pre- and postoperative nomograms for predicting the recurrence of patients with NBNC-HCC after resection. These two easy-to-apply graphical models will be valuable in guiding preoperative treatment planning, adjuvant treatment implementation, and postoperative monitoring for patients with NBNC-HCC.

## Data Availability Statement

The raw data supporting the conclusions of this article will be made available by the authors, without undue reservation.

## Author Contributions

KL, QH, LW, WZ, and JL: study conception, design, quality control of this study, drafting of the manuscript, and statistical analysis. JZ, ZD, HL, JF, PG, and ZC: data acquisition and data interpretation. YZ, WZ, and JL: resource and study supervision. All authors contributed to the article and approved the submitted version.

## Funding

This study was supported by Science and Technology project of Fuzhou (Grant number: 2019-SZ-49), Key Clinical Specialty Discipline Construction Program of Fuzhou, Fujian, P.R.C (Grant number: 201912002) and Fujian Provincial medical center of hepatobiliary.

## Conflict of Interest

The authors declare that the research was conducted in the absence of any commercial or financial relationships that could be construed as a potential conflict of interest.

## Abbreviations

NBNC-HCC, Non-B non-C hepatocellular carcinoma; NAFLD, non-alcoholic fatty liver disease; ALD, alcoholic liver disease; BCLC, Barcelona Clinic Liver Cancer staging system; HBcAb, hepatitis B virus core antibody; RBCs, red blood cells; WBCs, white blood cells; Hb, hemoglobin; ALT, alanine aminotransferase; AST, aspartate aminotransferase; GGT, γ-glutamyl transpeptidase; NLR, neutrophil to lymphocyte ratio; PLR, platelet to lymphocyte ratio; PNI, prognostic nutritional index; ANRI, aspartate aminotransferase to neutrophil ratio index; ALRI, aspartate aminotransferase to Lymphocyte ratio index; PNLR, platelet times neutrophil to lymphocyte ratio; GPR, γ-glutamyl transpeptidase to platelet ratio; NγLR, neutrophil times γ-glutamyl transpeptidase to lymphocyte ratio; DNLR, derived neutrophil to lymphocyte ratio; AFP, alpha fetoprotein; MVI, microvascular invasion.
