# Supplementary material for: Pre- and Postoperative Models for Prediction of Recurrence in Non-B, Non-C Hepatocellular Carcinoma
Source: Front Oncol. 2021 Feb 18;11:612588. doi: 10.3389/fonc.2021.612588 (PMC7930483; doi:10.3389/fonc.2021.612588)
Supplement: Supplementary file 1 [file DataSheet_1.docx]

# Supporting information

**Pre- and postoperative models for prediction of Recurrence in Non-B, Non-C Hepatocellular Carcinoma**

**Related Computerized Programs for Nomogram With R**

library(rms)

**For Cox Proportional Hazards Model**

f <- cph(Surv(RecurrenceT,Recurrence==1)~Tumor_Number…., x=T, y=T, data= training_cohort, surv=T)

**For Nomogram**

surv <- Survival(f)

surv1 <- function(x)1-surv(1,lp=x)

surv2 <- function(x)1-surv(2,lp=x)

surv3 <- function(x)1-surv(3,lp=x)

nom<-nomogram(f, fun=list(surv1, surv2, suvr3), lp = F, funlabel=c('1-Year Recurrence probability', '2-Year Recurrence probability', '3-Year Recurrence probability'), maxscale=100, fun.at = c('0.9','0.85','0.8','0.7','0.6','0.5','0.4','0.3','0.2','0.1'))

**Plot Nomogram**

plot(nom, cex.axis =1.0,cex.var=1.0)
